# Behçet's Disease, Associated Large Vessel Thrombosis, and Coexistent Thrombophilia: A Distinct Nosological Entity?

**DOI:** 10.1155/2013/740837

**Published:** 2013-06-23

**Authors:** Dimitrios Stoimenis, Nikolaos Petridis, Nikos Papaioannou

**Affiliations:** 1st Department of Internal Medicine, “Georgios Papanikolaou” General Hospital, Exochi, 570 10 Thessaloniki, Greece

## Abstract

Behçet's disease (BD) represents a multisystemic disorder that combines features of immune-mediated diseases and autoinflammatory disorders. Even though it is recognized that every type or size of vessel can be affected in this disease, there is an inability to describe a coherent model that sufficiently explains the predilection of certain patients with BD for manifesting severe large vessel thrombosis. The inconsistent epidemiologic data and the complex genetic background of BD, along with the controversy of multiple international studies regarding the coexistence of thrombophilia in patients with BD and large vessel thrombosis, make us think that a percentage of these patients may actually suffer from a distinct clinical entity. The stimulus for this concept arose from the clinical observation of three male patients who were admitted to our clinic due to extended vena cava thrombosis. On the occasion of those clinically and laboratory resembling cases, we performed a literature review concerning the epidemiology of BD, associated thrombosis, and coexistent thrombophilic factors, in order to present some evidence, which sustains our hypothesis that certain patients with large vessel thrombosis, who share features of BD and coexistent thrombophilia, should actually be further investigated for the possibility of suffering from a distinct nosological entity.

## 1. Introduction

Behçet's disease (BD), which is also acknowledged as Adamantiades-Behçet's disease, is classically defined as a chronic multisystemic vasculitis affecting vessels of any type or size and it is mostly characterized by recurrent mucocutaneous and ocular lesions. Over the last few years, the studies conducted on the pathophysiology of BD demonstrated its common characteristics with the group of autoinflammatory diseases. As a result, nowadays, many authors prefer to use an alternative term that defines BD as a chronic, relapsing, and multisystem autoinflammatory disorder of unknown etiology [1]. Despite the international concern and the copious researches on BD, no pathognomonic laboratory finding or diagnostic test has yet been validated and the diagnosis of the disease remains a clinical decision. Over 15 different sets of criteria for the diagnosis of BD have been proposed but the most commonly used are the criteria of the International Study Group (ISG) which were created in 1990 [2] and the Japanese criteria that were revised by the Behçet's Disease Research Committee of Japan in 2003 [3, 4].

According to the ISG criteria, the diagnosis of BD requires the presence of recurrent oral ulceration plus any two of genital ulceration, typical defined eye lesions, typical defined skin lesions, or a positive pathergy test (Table 1). In one study of pediatric BD, the average period length between the initial oral ulceration and the second major manifestation was 8.8 years [17]. The Japanese criteria are divided into major and minor manifestations and were created in order to avoid overdiagnosis of BD. The disease is classified as complete, incomplete, possible, or specific type, based on the combination of those manifestations (Table 2). The ISG criteria emphasize the oral aphthosis and a positive pathergy test, but they do not consider other important site-specific manifestations such as the vascular lesions, which in certain cases can be the initial presentation of BD [18]. On the other hand, the Japanese criteria embody most of the site-specific manifestations, denominating three particular types of BD (neuro-, intestinal-, and vasculo-BD), but the diagnosis of the complete type of BD requires the presence of all the 4 predominant manifestations of BD. Therefore, although both sets of diagnostic criteria provide a useful diagnostic tool for the clinicians, they cannot ensure case ascertainment.

BD presents a significant inhomogeneity in clinical features, in the reported prevalences of the specific types, in gender predominance, and in genetic and immunological findings, vastly depending on which geographic region or ethnicity is studied. Thereafter, the inconsistent presentation of BD raises the question whether this is the result of a multifactorial interaction or if there is a possibility that what we call Behçet's disease is to be constituted by a group of overlapping separate diseases. In this paper, we initially describe three cases with major vein thrombosis from which the first was diagnosed as complete vasculo-BD, the second was diagnosed as possible vasculo-BD, and the third was diagnosed as deep vein thrombosis (DVT) due to multifactorial thrombophilia. On the occasion of those three clinically and laboratory resembling cases, we performed a literature review concerning the epidemiology of BD, associated thrombosis, and coexistent thrombophilic factors, in order to support our hypothesis that BD might not represent a unique clinical entity and that in certain patients with large vessel thrombosis, who present some features of BD and coexistent thrombophilic factors, the possibility of suffering from a distinct nosological entity should be further investigated.

## 2. Subjects and Methods

Three cases with vena cava thrombosis were studied. Demographic characteristics, clinical signs and symptoms, radiologic imaging, laboratory and thrombophilic testing, treatment, and outcome were recorded. 

## 3. Results

Three adult Caucasian males of Greek origin aged 32, 54, and 20 (patients A, B, and C, resp.) were admitted to our Department of Internal Medicine due to large vessel thrombosis. All of the three patients had free medical history.

### 3.1. Clinical, Laboratory, and Imaging Assessments

**Figure 1:**
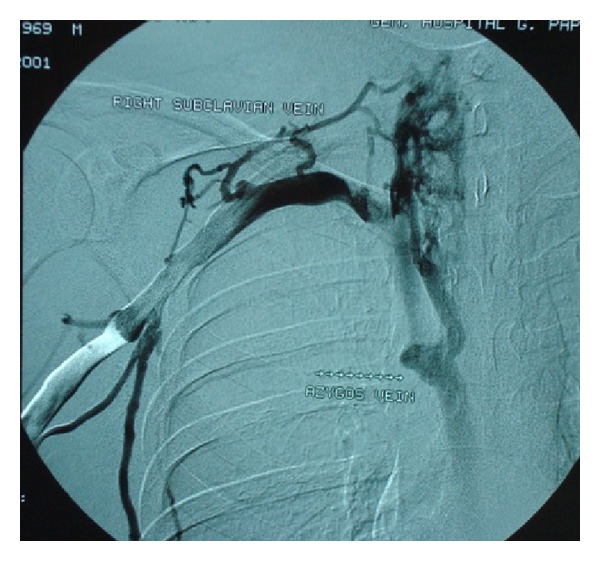
Digital subtraction angiography (iv-DSA) of superior vena cava (SVC) in patient A showing the extended SVC and right subclavian vein thrombosis along with the establishment of collateral flow through the azygos vein.

**Figure 2:**
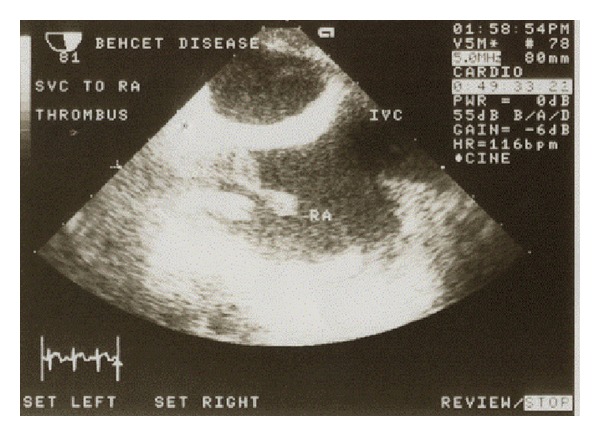
Transesophageal echocardiogram (Triplex) in patient A showing a sizeable (3 cm) pedunculated thrombus, which was oscillating in the right atrium (RA).

*Patient A.* A 32-year-old male was admitted to our department with complaints of dizziness, headache, shortness of breath, and persisting low-grade fever starting a month ago. On initial evaluation, the patient had facial edema and distended jugular and upper chest veins. Additionally, tongue, gingival, and scrotal aphthous ulcers were observed. The patient confirmed the presence of recurrent oral aphthosis the last semester, which was painful and self-limited. A nodular episcleritis of the left eye was observed during ocular examination and pathergy test was positive, while no other cutaneous manifestations were observed. Digital subtraction angiography revealed an extended thrombosis of the superior vena cava (SVC) and the right subclavian vein and the creation of a collateral pathway through the azygos vein (Figure 1). Further evaluation with a transesophageal echocardiogram (triplex) showed a sizeable (3 cm) pedunculated thrombus, which was oscillating in the right atrium (Figure 2). Laboratory findings were characterized by moderately elevated inflammatory markers and mild normochromic anemia, while the immunological tests identified the HLA-B*51 haplotype and enhanced C4 complement titer, with negative results for the autoantibodies testing (Table 3). The patient was assessed for thrombophilic factors and was found to be homozygous carrier for the C677T polymorphism of the 5,10-methylene-tetrahydro-folate reductase (MTHFR) mutation, but serum homocysteine was within the normal range (Table 4).

**Figure 3:**
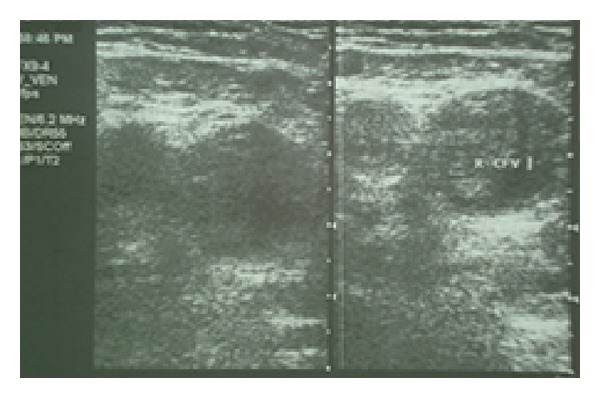
Triplexultrasound in patient B showing the thrombosis of the right common femoral vein (CFV). The vein is uncompressible.

*Patient B.* A 54-year-old male was referred to our department from a vascular surgery clinic due to a recently diagnosed inferior vena cava thrombosis. Patient's history was remarkable for a 7-month bilateral and gradually ascending deep vein thrombosis of the lower limps; despite the fact that ever since he was diagnosed with the initiatory left popliteal vein thrombosis, he was treated with low molecular weight heparin (LMWH), used at therapeutic dose. Clinically, the patient had painful aphthous ulcers on the lips, gingiva, and oral cavity, characterized by frequent recurrence over the past 2 years. An ocular test was unremarkable; however, the pathergy test was positive although no other cutaneous lesions were observed. The patient had had triplex ultrasound repeated over the last months, which showed the bilateral and scaling course of the thrombosis of the large vessels of the lower limps, the iliac and the femoral veins (Figure 3), and the inferior vena cava. A computed tomography scan with IV contrast confirmed the sonographic findings. Laboratory assessment revealed elevated inflammatory markers and C3 complement titer, mild normochromic anemia, and positive HLA-B*51 type, while the results of the autoantibodies were all negative (Table 3). Moreover, the patient was found to be positive in several thrombophilic factors as he was heterozygous carrier of MTHFR C677T and FV-Leiden R506Q mutations with associated severe hyperhomocysteinemia (Table 4).

**Figure 4:**
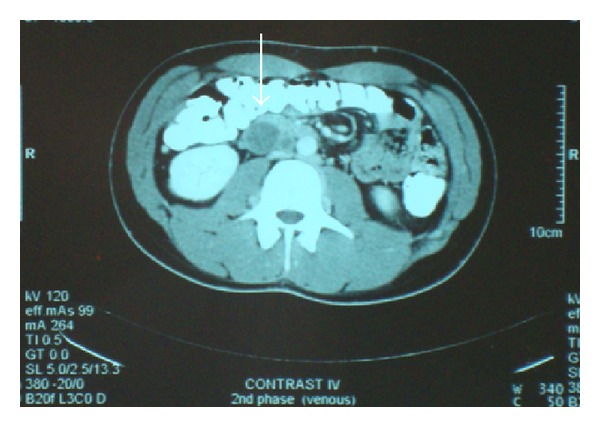
Computed tomographic angiography in patient C showing the thrombosis of the inferior vena cava along with a significant degree of perivascular inflammation.

*Patient C.* A 20-year-old male was admitted to our department for a three-week continuous low back and thigh pain, accompanied by local tenderness, warmth and erythema, and a two-week fever. One month before his admission, the patient had been hospitalized in a vascular surgery clinic, where the diagnosis of inferior vena cava thrombosis was made, with an expansion of the thrombus to the ileac and the femoral veins bilaterally. He had been discharged with LMWH, prescribed at therapeutic dose. On examination, he was febrile up to 38.3°C and tenderness was present in iliac fossa and thigh bilaterally and especially left, with no other notable findings. The oral cavity and scrotum were free of any aphthous or herpetiform ulcers and cutaneous manifestations were absent; however, the patient mentioned the occurrence of two episodes of painless, self-limited aphthous ulcers in the last 9 months. An ocular test was unremarkable and the pathergy test was negative. A CT angiography was performed showing unchanged thrombosis of the inferior vena cava, ileac, spermatic, and femoral vein and the creation of a collateral pathway, along with a significant degree of perivascular inflammation (Figure 4). Laboratory exams revealed highly elevated inflammatory markers, slightly enhanced IgA and C4 complement titer, mild normochromic anemia, and prolonged partial thromboplastin time as well as the identification of HLA-B*51 and B*27 haplotypes (Table 3). Screening for autoantibodies was negative. Thrombophilia testing showed a positive low titer lupus anticoagulant (LAC) and a borderline hyperhomocysteinemia, while the patient was also found to be heterozygous carrier of MTHFR C677T and FV-Leiden R506Q mutations (Table 4).

### 3.2. Differential Diagnosis

In all the three cases, no evidence of infection, an underlying malignancy, or any other systemic vasculitis was documented by the clinical, imaging, and laboratory assessments. The main diagnostic findings of the three patients are summarized in Table 5. 

*Patient A.* The occurrence of recurrent oral aphthosis, genital ulceration, and a positive pathergy test established the diagnosis of BD according to the ISG criteria. It is worth noting that his ocular finding (unilateral nodular episcleritis) has been reported as a rare BD finding but it is not included in the eye lesions in the diagnostic sets of criteria for BD. 

*Patient B.* Hisclinical assessment included recurrent oral aphthosis and a positive pathergy test, which could not establish Behçet's diagnosis according to the ISG criteria, but the implementation of the Japanese criteria of 2003 could not exclude a Behçet's diagnosis in this case. HLA-B*51 positivity, acute phase response, and predominantly the deteriorating vascular lesions, even though he was receiving antithrombotic therapy with LMWH at therapeutic dose, supported the diagnosis of a possible type of BD. The main differential diagnosis in this patient was paraneoplastic thrombosis but the failure of the 7-month LMWH therapy in combination with the extended CT scanning and the gastrointestinal endoscopy did not provide evidence of any underlying malignancy. Likewise, it is doubtful whether the coexistence of three thrombophilic factors can itself explain his deteriorating clinical course, considering he was under sufficient antithrombotic therapy.

*Patient C.* His diagnostic assessment was remarkable for the coexistence of several different thrombophilic factors. A possible diagnosis of antiphospholipid syndrome was assumed but the reexamination in 6 and 12 weeks later did not detect the lupus anticoagulant. From the clinical assessment, even though he mentioned recurrent oral aphthosis, this manifestation was not observed by any physician and could not be evaluated. His laboratory findings were noticeable for the acute phase response and positivity in HLA-B*51&B*27, which, combined with the intense perivascular inflammation in CT angiography, posed the question whether all these findings were the result of the multifactorial thrombophilia and the extended thrombosis alone or if another inflammatory disorder was present. A biopsy of the small saphenous vein was performed and the pathology report described normal findings with no presence of inflammation or clot formation. The patient was also tested for JAK mutations with negative results.

### 3.3. Treatment and Outcome

*Patient A.* He was treated with oral immunosuppressants (methylprednisolone plus azathioprine) and anticoagulant agents (LMWH was administered at therapeutic dose for 3 days, followed by acenocoumarol with an INR target of 3–3.5). A successful surgical removal of the right atrium thrombus was performed, along with an SVC and brachiocephalic vein biopsy. The pathology report of those specimens described clot in incipient organization, inflammatory, and reactive lesions, findings which were compatible with vasculitis. He was discharged with a prescription of oral azathioprine, methylprednisolone with tapering dosage, aspirin, acenocoumarol, folic acid, and colchicine only during the occurrence of oral ulcerations. Clinical improvement was obtained in 1 month with a decrease of the inflammatory markers within the normal range. In a period of 6 months, the thromboses partially subsided, with complete resolution of the thrombi and vessel recanalization in 24 months. No signs of a relapse were observed during the 11-year follow-up, apart from the sporadic oral aphthosis.

*Patient B.* On admission, LMWH was discontinued and he was given acenocoumarol therapy with an INR target of 3–3.5. Soon after the completion of the initial diagnostic procedures, oral immunosuppressants (methylprednisolone plus azathioprine), colchicine, and folic acid per os were added in his medication. One month later, significant clinical improvement was observed with a decrease in the inflammatory markers within normal range. He was discharged with a prescription of oral azathioprine, methylprednisolone with tapering dosage, acenocoumarol, folic acid, and colchicine only during the occurrence of oral ulcerations. CT angiography of the IVC was performed in the 2-month follow-up revealing considerable remission of the thromboses with sufficient blood flow and partial recanalization of the IVC and the iliac veins. Complete clinical remission was observed in the total 6-month follow-up.

*Patient C.* He was treated with anticoagulant agents, which included LMWH at therapeutic dose followed by oral acenocoumarol with an INR target of 3–3.5. Folic acid was also added to his medication. Patient's inflammatory markers gradually decreased within normal range, and the fever subsided during his hospitalization without any anti-inflammatory medication. A CT angiography prior to his discharge showed the subsidence of the perivascular inflammation but no improvement of the thrombosis. Patient's assessment did not confirm any of the hallmark manifestations of BD, and he was discharged with a prescription of oral acenocoumarol. In the total 7-month follow-up, the patient presented distention of the abdominal veins due to the IVC obstruction, while his repeated imaging assessment, which included ultrasonography, computed tomography angiography, and magnetic resonance venography, has not shown sufficient subsidence of the DVT and IVC thrombosis. During the follow-up, examination for lupus anticoagulant was negative and no sign of any underlying malignancy or systemic vasculitis was evidenced from the elaborate clinical, laboratory, and imaging assessment.

## 4. Discussion

### 4.1. Epidemiology of Behçet's Disease

Although there is evidence for a global incidence, BD is more prevalent in the Far East, Middle East, and Eastern Mediterranean basin, following the pattern of the old silk trading routes, whereas it is rarely encountered in Northern Europe, Northern Asia, Sub-Saharan Africa, and America [19]. The highest prevalence of BD is observed in Turkey with 420 cases per 100,000 population, while reported estimates vary from 13.5–22 cases per 100,000 population in Japan, Korea, China, Iran, and Saudi Arabia [20], and between 0.12 and 7.5 per 100,000 population in Europe and the US [21]. However, the epidemiology cannot be scaled with accuracy, since the worldwide reported prevalences of BD are strongly influenced by the miscellaneous diagnostic sets of criteria that are applied in every country. There is a general estimation that BD occurs roughly equally in both sexes, with a worse prognosis and a more severe course of the disease in men [19]. Nevertheless, there are several reports showing a divergent gender predominance of BD [20, 21]. In the Middle East, there are reports indicating a male predominance of BD, with male-to-female ratios of 3.8 : 1 in Israel and 5.3 : 1 in Egypt [20]. Quite the opposite, in the United States there is a predominance of the females, with a female-to-male ratio of 5 : 1, whilst in Germany, Japan, and Brazil, the disease is slightly more common in females [20]. As far as the age of BD onset is concerned, the symptoms typically occur in the late third and early fourth decades of life [20]. Juvenile BD is rather unusual, representing 3% to 7% of all cases [22].

The pathogenesis of the disease remains uncertain. It is believed that genetic, autoimmune end environmental factors interact with each other. Historically, the most consistent association is with HLA-B*51 gene (chromosome 6p21) although the third of BD patients do not possess this gene [23]. The geographic distribution of this gene is concomitant to the prevalence of BD in specific latitudes, namely, between 30° and 45° North [23]. In particular, in countries in the southern hemisphere and in Europe above 45° North the prevalence of HLA-B*51 in healthy individuals is nil or low (1–10%), in countries in Central Europe its prevalence is intermediate (11–15%), and in countries in the eastern Mediterranean basin and in Middle East its prevalence is high (>15%) [23]. Other MHC associations with BD include tumor necrosis factor (TNF), the heat shock protein (HSP) family, and the MHC class I chain gene A (MICA) [23]. Moreover, 16 different non-HLA susceptibility loci were identified in whole-genome screening as being associated with the disease [24]. The environmental factors that have been most implicated in triggering the inception of BD are infectious agents such as Herpes simplex virus type 1 (HSV-1), *Streptococcus sanguinis, S. oralis, S. mitis*, and* Saccharomyces cerevisiae* [19, 23, 25].

The most intriguing feature in the epidemiologic study of BD concerns the regional variation of its clinical expression, especially regarding the neurological, vascular, and intestinal manifestations of BD. In the case of neuro-BD, the reported percentages range from 1.3% to 59% in hospital-based series and 5.3% to 14.3% in three prospective studies from Turkey, Iran, and Iraq [26]. Similarly, gastrointestinal involvement in BD has been frequently reported in Japan (50–60%), United Kingdom (38–53%), and Taiwan (32%), whereas it is rare in Turkey (3%), Saudi Arabia (4%), and Jordan (5%) [27]. Lastly, the same disparity is presented in the reported prevalences of vascular BD, which is frequent in Saudi Arabia (34%) [28] and Turkey (14.3–26%) [29, 30] and lower in Iran (8.3%) [31] and Japan (6%) [32]. However, we must notice that the lack of case definition can partially explain the deviant reported prevalences. For instance, in the case of vascular BD, there is no consensus concerning the lesions that should be included in the clinical vascular characteristics of BD. Therefore, in Japan, patients with superficial thrombophlebitis are excluded from studies that examine the frequency of vascular involvement in BD, since this lesion is categorized as a skin manifestation according to the Japanese criteria.

### 4.2. Epidemiology of Vasculo-Behçet's Disease and Coexistent Thrombophilic Factors

The term “vasculo-Behçet,” which is included in the specific types of BD according to the Japanese criteria of 2003, is used in order to identify that subcategory of the patients with BD who have a tendency to develop large vessel lesions, often with an additive and progressive course [33]. The frequency of the vascular involvement among patients with BD ranges from 7.7 to 38% [18]. The lesions involve mainly the veins (29% of BD cases) and less frequently the arteries (8–16% of BD cases) and include vasculitis, thrombosis, and aneurysms [34]. Thrombosis represents the most usual manifestation, whereas the rupture of a pulmonary artery aneurysm consists the leading cause of death in vasculo-BD [33]. Nevertheless, the precise worldwide prevalences of large vein thrombosis in BD could not be recorded in this literature review because the majority of the international studies examine the large vessel involvement as a whole and they do not use a common and clear case definition. In contrast, cardiac involvement with clinical implication is rather unusual, since the reported incidence range from 1% to 5% [35]. Cardiac manifestations mainly include coronary aneurysms, pericarditis, endocarditis, myocarditis, and predominantly right-sided intracardiac thrombosis [34].

The pathophysiology of vascular thrombosis in BD is not well understood. It is generally accepted that the triggering feature in the thrombogenesis process is the vascular inflammation. However, the presence of large vessel thrombosis only in certain patients with BD implies that other additional factors are required for the development of a thrombotic event. La Regina et al. proposed that thrombogenesis in BD can be explained through the concept of Virchow's triad of venous thrombosis (abnormal blood flow, abnormal vessel wall, and abnormal blood constituents) [36].

Several abnormal blood constituents have been implicated in the thrombogenesis in BD, with an emphasis given in certain procoagulant factors (factor V Leiden and prothrombin mutations, MTHFR polymorphisms, hyperhomocysteinemia, and antiphospholipid antibodies), but the results of the related researches are controversial (Table 6); Gül et al. found a positive association between FV Leiden and FII G20210A mutations with venous thrombosis in BD in Turkish patients [8, 9], whereas Silingardi et al. did not confirm these results in Italian patients [10]. Karakus et al. reported that homozygosity of MTHFR C677T was significantly higher in BD patients than in healthy controls in Turkish patients [11], but Ricart et al. did not find such an association in patients from eastern Spain [12]. Shahram et al. found significantly higher homocysteine levels in Iranian patients with BD and thrombosis [13], whereas Leiba et al. found homocysteine levels within normal range among Israeli patients with BD and thrombosis [14]. Lastly, no association was found between anticardiolipin antibodies titres and vascular involvement in BD in Turkish patients by Tokay et al. [15], while statistically significant presence of aCL was reported in patients with BD in an Italian study by Hull et al. [16].

All these conflicting data get even more perplexing if we further examine the factor V Leiden (FVL) and prothrombin 20210A mutations. FVL is mainly encountered in Caucasians, with an average prevalence of 5%, and increases the risk of venous thrombosis 3- to 8-fold for heterozygous carriers and 50- to 80-fold for homozygous carriers [5]. The Mediterranean region presents the highest prevalence of FVL in the world, while FVL is very rare or absent in non-Caucasian ethnic groups (Africans, South-East Asians, Chinese, Japanese, American Indians, Greenland Eskimos, and Aboriginals of Australia) [7]. Likewise, FII 20210A mutation is met only in Caucasians with an average prevalence of 2-3% and increases the risk of venous thrombosis 3-fold [5]. The highest prevalence of FII 20210A is encountered in the Southern Europe, in the Mediterranean region and in the Middle East [10, 37].

 These epidemiologic data point out that the highest prevalences of both these inherited procoagulant factors are quite proportional to the highest epidemiologic prevalences of vasculo-BD, posing the question whether it is a coincidental coexistence or if there is a genetic association. Trying to answer this question, we examined random studies from literature review concerning the association between BD, FVL, and FII 20210A mutations. As we have noticed previously, FVL and FII 20210A are absent in Japan but prevalent in Turkey (*p* = 7.41% and 0.7–8.0%, resp.) and in Lebanon (*p* = 14.4% and 1.3–3.6%, resp.) [37, 38]. Assuming that the reports of Gül et al. [8, 9] indicate that FVL and FII 20210A mutations are genetically associated with BD and act synergistically with the tenderness of BD for thrombotic events, one could say that this is one reason that explains why the prevalence of vascular BD is lower in Japan (*p* = 6%) [32] and higher in Turkey (*p* = 14.3–26%) [29, 30] and in Lebanon (*p* = 36.8%) [39]. Consequently, we should expect that this correlation applies in Italy and in Spain as well, where FVL and FII 20210A mutations are also frequently encountered; nevertheless, the studies from Silingardi et al. [10] and Espinosa et al. [40] did not find such a positive association between those thrombophilic mutations and BD in Italian and Spanish patients, respectively. 

### 4.3. Epicrisis

The main argumentations for the inconsistency of clinical, laboratory, and genetic findings in BD are the multifactorial interaction in the pathogenesis of the disease and the lack of case ascertainment, which in many patients result in an inappropriate diagnosis of a possible or an incomplete type of BD. Therefore, the worldwide studies may present a discrepancy in their results, on account of the fact that the experimental groups embody several subjects who actually do not suffer from BD. But then, in all these patients that present only certain features of BD, which is the nature of their actual disease? In a recently published paper, which was brought to our knowledge while writing this paper, Yazici et al. pose a similar questioning about the heterogeneity of BD and reach the conclusion that BD may actually represent more than one condition, underlining three more crucial parameters of BD [41]. The first is that a Mendelian inheritance pattern does not seem to exist among the adult patients, whereas an autosomal recessive inheritance pattern has been suggested for the pediatric patients. The second is that a differing response of different clinical manifestations is encountered to one same drug. The third and most essential parameter is that two discrete symptom clusters can be recognized in BD at the present time, the acne and arthritis cluster and the vascular cluster, in which, the dural sinus thrombosis of neuro-BD and the superficial vein thrombosis, the deep vein thrombosis, and the pulmonary artery involvement of vasculo-BD all seem to be clustered in BD patients [41].

In the aforementioned three male patients, clinical presentation was dominated from the extended vena cava thrombosis. Their laboratory assessment revealed elevated inflammation markers, HLA-B*51 positivity, and several coexisting thrombophilic factors. Their thrombophilia was not enough to interpret their deteriorating large vessel thrombosis considering the failure of the anticoagulant therapy to prevent the expansion of the thrombosis. The exclusion of infectious and malignant causes suggested the possibility of an underlying autoimmune or autoinflammatory mechanism as a contributory factor in the lasting thrombogenesis process. Despite the absence of typical for BD ocular manifestations, the diagnostic assessment showed that this factor was BD in patient A and most probably in patient B as well, taking into consideration his direct response to the use of immunosuppressant agents. In patient C the lack of definite oral ulceration and a negative pathergy test or other clinical manifestations led us in a diagnostic stalemate.

**Figure 5:**
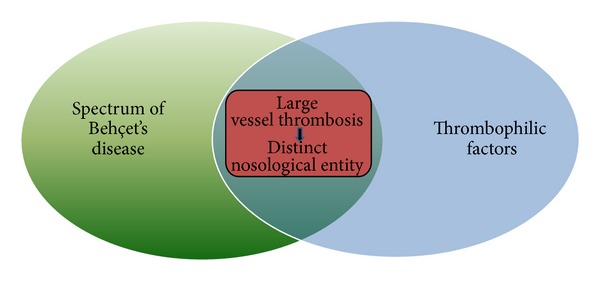
Our hypothesis of a distinct nosological entity evolves patients who present some features of the complex spectrum of BD and various thrombophilic factors. The synergistic epistasis in this pathologic pathway results in large vessel thrombosis of those patients.

The inconsistent and frequently contradictory features of BD, especially regarding vasculo-BD and coexistent thrombophilic factors that were presented previously, demonstrate the significant heterogeneity of this disease, leading us to the hypothesis that a lot of patients who are diagnosed as suffering from BD might not have the same disease but different overlapping clinical entities. We further assume that patients who present large vessel thrombosis, coexistent thrombophilic factors, and some BD features, for instance oral aphthosis and HLA-B*51 positivity, may actually suffer from a distinct nosological entity (Figure 5). In this clinical entity, the complex interaction among genetic loci could be explained with the phenomenon of epistasis. Epistasis, which is usually found in the human major histocompatibility complex genes, is a genetic term that was invented from William Bateson, who observed that, in some dihybrid crosses, not all possible phenotypic classes were observed and/or that some gene combinations resulted in novel phenotypes [42]. The concept of synergistic epistasis could theoretically answer the question why certain patients with BD have a specific tendency for large vessel thrombosis. Besides, the existence of such a distinct nosological entity, which shares some features of the complex spectrum of BD, could explain why certain only international studies confirm a significant association between BD and specific thrombophilic factors, as well as why the frequency of vascular thrombosis in BD is lower in Japan, where some factors such as V Leiden and II G20210A mutations are absent. 

Nevertheless, there is a range of limitations to this medical hypothesis that need to be acknowledged. The three cases presented in this paper comprise a negligible sample in order to reach definite conclusions. Furthermore, the relatively short duration of follow-up in patients B and C (less than a year) must be taken into consideration, since we cannot rule out the possibility that another known cause of thrombosis might be clinically evidenced in the forthcoming period. Besides, apart from the malignancies and the established immunological causes of thrombosis (systemic lupus erythematosus, inflammatory bowel disease, and BD), current evidence suggests that many other defined autoimmune disorders and immune-mediated diseases are closely associated with an increased risk of venous thromboembolism [43]. Finally, even if the hypothesis of this paper was verifiable, it still seems that this theoretical model of venous thrombosis could not explain every single case of large vessel thrombosis in BD, given that in certain international studies it is demonstrated that thrombosis in BD can occur in spite of the absence of—at least all identified—thrombophilic factors [12, 14, 40].

Consequently, it is unquestionable that our hypothesis for the potential existence of a distinct nosological entity, which entails a genetic linkage between thrombophilia and a novel inflammatory condition that shares certain features of BD, cannot be documented only by the aforementioned cases and the analysis of the preexisting literature. Further studies are required, in order to determine if there is indeed a subgroup of patients with large vessel thrombosis, coexistent thrombophilia, and poor response to the anticoagulant therapy and at the same time they do present certain other common clinical manifestations and laboratory findings of BD. Moreover, the thrombotic phenomenon in these distinct overlapping clinical entities may emphasize the necessity of studying the coagulation physiology in combination with the immune response as it is studied in basic sciences [44]. Recently, the term immunothrombosis has been suggested in order to describe a physiological form of thrombosis that supports innate immune defence in microvessels [45]. The use of the term immunothrombosis could be suitable in order to describe thrombotic phenomena with immunological background in large vessels if any correlation is evidenced.

## 5. Conclusions

Epidemiologic data and varied genetic background suggest that BD might not constitute a single disease. Moreover, in a number of patients with large vessel thrombosis, coexistent thrombophilia, acute phase response, lack of malignancy or other known causes of thrombosis, and failure of the antithrombotic therapy alone to treat or at least prevent the expansion of the thrombosis, it seems that another inflammatory disorder is required in order to trigger the thrombogenesis process and therefore the basis for their treatment is immunosuppressant agents. In these cases that share various common features with BD, it is uncertain if the dilemma of the proper treatment, anticoagulant, and/or immunosuppressive treatment can be answered through the heterogeneous spectrum of BD. Distinguishing such patients as suffering from a distinct nosological entity is a hypothesis that can be proved only with further studies. On the contrary, if our hypothesis is incorrect and these patients do represent cases of BD with coincidental coexistent thrombophilia, taking into consideration the proneness of BD to thrombotic events and the highest concomitant epidemiologic prevalences of vasculo-BD and specific inherited procoagulant factors, we propose that all patients with BD who manifest thrombotic events should be assessed for thrombophilia testing, especially those living in Southern Europe, in the Mediterranean basin, and in the Middle East, so that their optimal management can be appropriately weighed. 

## Figures and Tables

**Table 1:** Diagnostic criteria of the International Study Group for Behçet's disease (1990).

Criteria	Definition
Recurrent oral ulceration	Minor aphthous, major aphthous, or herpetiform ulceration observed by physician or patient, which recurred at least 3 times in one 12-month period.
*Plus any two of *	
Recurrent genital ulceration	Aphthous ulceration or scarring, observed by physician or patient.
Eye lesions	Anterior uveitis, posterior uveitis, or cells in vitreous on slit lamp examination or retinal vasculitis observed by ophthalmologist.
Skin lesions	Erythema nodosum observed by physician or patient, pseudofolliculitis or papulopustular lesions, or acneiform nodules observed by physician in postadolescent patients not on corticosteroid treatment.
Positive pathergy test	Papule/Pustule ≥2 mm surrounded by an erythematous halo, 24–48 h (read by physician) after a blunt needle prick (18–21 gauge) on the intradermal and subcutaneous part of the volar forearm at an angle of 45° to a depth of 5 mm.

**Table 2:** Diagnostic criteria of the Behçet's Disease Research Committee of Japan (2003 revision).

Criteria	Diagnosis
Major manifestations: (1) *Recurrent oral aphthous ulceration* (2) *Skin lesions* (i) Erythema nodosum (ii) Subcutaneous thrombophlebitis (iii) Folliculitis, acne-like lesions (iv) Cutaneous hypersensitivity (3) *Eye lesions* (i) Iridocyclitis (ii) Chorioretinitis, retinouveitis (iii) Definite history of chorioretinitis or retinouveitis (4) *Genital ulcers* Minor manifestations (1) *Arthritis without deformity and ankylosis* (2) *Gastrointestinal lesions characterized by ileocecal ulcers* (3) *Epididymitis* (4) *Vascular lesions* (5) *Central nervous system symptoms *	(I) Complete type *Four major manifestations appear in the course of the illness. *
	(II) Incomplete type (A) *Three major manifestations or 2 major and 2 minor manifestations appear in the course of the illness.* (B) *Typical ocular manifestations and one major or two minor manifestations appear in the course of the illness. *
	(III) Possible type *Certain major manifestations appear but do not qualify as the incomplete type and typical minor manifestations repeat or worsen. *
	(IV) Specific types (a) *Intestinal-BD* (b) *Vasculo-BD* (c) *Neuro-BD *
	*Additional assessment for reference (not mandatory)* (i) Positive/negative pathergy test. (ii) Positive/negative prick test with a streptococcus vaccine. (iii) Inflammatory response (ESR, CRP, WBC, Complement titer). (iv) Positive/negative HLA-B*51. The type of HLA type should be listed. (v) Pathologic findings.

**Table 3:** Laboratory findings of our patients at presentation.

Laboratory value	Patient A	Patient B	Patient C	Normal range
Hematocrit	**36.5**	**38.1**	**38.3**	40.0–52.0%
Hemoglobulin	**12**	**12.1**	**12.3**	13–17.8 g/dL
MCV	90	81.8	83	80−99 fL
Leukocytes	8.60	9.83	9.80	4.0−10 × 10^3^/*μ*L
Neutrophils	59	74	74.2	40−75%
Lymphocytes	32	14.1	21.3	20−45%
Platelets	262	**513**	304	150−450 × 10^3^/*μ*L
PT	11	61.4*	14.1	10.5–12.5 sec
apTT	30	39.3*	**52.5**	27–34 sec
d-dimers	0.1	**1.0**	**2.6**	0–0.3 *μ*g/mL
Fibrinogen	3.88	**5.8**	**5.8**	2–4 g/L

*patient B was on acenocoumarol therapy when tested				
Total Proteins	7.9	7.20	8.10	5.5–8 g/dL
Albumin	4.6	**3.40**	4.20	3.5–5.5 g/dL
Globulins	3.3	3.80	3.90	1.5–3.5 g/dL
ESR	**38**	**52**	**80**	1−10 mm/1rst hour
C-reaction Protein	**3.55**	**9.54**	**10.10**	<0.80 mg/dL
IgA	171	409	**501**	85−450 mg/dL
IgG	**1736**	958	1136	800−1700 mg/dL
IgM	170	**37**	**57**	63−277 mg/dL
C3 complement	170	**213**	126	85–193 mg/dL
C4 complement	**53**	33.6	**41**	12–36 mg/dL
RF	<15	<15	<15	0–20 IU/mL
ANA	Negative	Negative	Negative	
Anti-DNA	Negative	Negative	Negative	
P-ANCA	Negative	Negative	Negative	
C-ANCA	Negative	Negative	Negative	
Anti-ENA	Negative	Negative	Negative	
HLA type	Β*51	Β*51	Β*51-B*27	
R.P.R.	Negative	Negative	Negative	
HBsAg	Negative	Negative	Negative	
IgM anti-HBc	Negative	Negative	Negative	
Anti-HCV	Negative	Negative	Negative	
H.I.V Ag/Ab Combo	Negative	Negative	Negative	

**Table 4:** Results of the thrombophilia testing in our patients.

Laboratory value	Patient A	Patient B	Patient C	Normal range
Antithrombin III	92	162	76	70–120%
Protein C	102	80	88	70–140%
Protein S	85	87	117	70–140%
Anticardiolipin IgG	6.9	8.5	9.6	<20 GPL
Anticardiolipin IgM	16.5	14	13.6	<20 MPL
aB2GPI IgG	2.2	4.1	4.3	<20 GPL
aB2GPI IgM	14.5	11.7	12	<20 MPL
Lupus Anticoagulant	1.02	1.07	**1.44**	<1.20
PCR FV Leiden	Negative	**Heterozygous** **R506Q**	**Heterozygous** **R506Q**	
PCR FII20210A	Negative	Negative	Negative	
PCR MTHFR	**Homozygous** **C677T**	**Heterozygous** **C677T**	**Heterozygous** **C677T**	
Homocysteine	12.2	**26**	**15**	<15 *μ*mol/L
F VIII	92	102	107	60–150%
F IX	70	75	79	60–150%

**Table 5:** Synopsis of the three patients' diagnostic assessment.

	Cardiovascular thromboses	Thrombophilic factors	HLA	ISG criteria 1990	Japanese criteria 2003
Patient A 32-year old	(i) SVC (ii) Subclavian vein (right) (iii) Intracardiac (right atrium)	(i) Homozygous MTHFR C677T	B*51 Positive	(1) Recurrent oral aphthosis + (2) Genital ulceration + (3) Positive pathergy = **Behçet's Disease**	(1) Verifiable recurrent oral aphthosis + (2) Genital ulceration + (3) Vascular lesions = **Possible BD** (i) Positive pathergy (ii) Vasculitic lesions in SVC and brachiocephalic vein biopsy

Patient B 54-year old	(i) IVC (ii) Iliac veins bilaterally (iii) Femoral veins bilaterally (iv) Deep veins of lower limbs	(i) Heterozygous MTHFR C677T (ii) Hyperhomocysteinemia (iii) Heterozygous FV Leiden R506Q	B*51 Positive	(1) Recurrent oral aphthosis + (2) Positive pathergy = **Insufficient criteria for diagnosis of BD**	(1) Verifiable recurrent oral aphthosis + (2) Deteriorating vascular lesions = **Possible BD** (i) Positive pathergy

Patient C 20-year old	(i) IVC (ii) Iliac veins bilaterally (iii) Femoral veins bilaterally (iv) Spermatic vein (v) Deep vein of lower limbs	(i) Heterozygous MTHFR C677T (ii) Hyperhomocysteinemia (iii) Heterozygous FV Leiden R506Q (iv) Transitory positive Lupus Anticoagulant	B*51 B*27 Positive	**Lacking criteria for diagnosis of BD**	(1) Reported episodes of painless oral aphthosis, not observed by any physician + (2) Deteriorating vascular lesions = **Lacking criteria for diagnosis of BD ** (i) Negative pathergy (ii) Normal pathologic findings in small saphenous vein biopsy

**Table 6:** Prevalence of the procoagulant factors in the general population, in patients with BD, and in patients with BD and thrombosis.

Thrombophilic factors	Prevalence in the general population [5–7]	Prevalences of each thrombophilic factor in studies from literature review [8–16]		
		All patients with BD	Patients with BD and thrombosis	Controls patients
FV leiden mutation	5% in Caucasians (range: 0–15%) Rare or absent in black African and Far East Asian people.	Gül et al. [8] 23% (15/64)^†^, *Turkish patients*	Gül et al. [8] 37.5% (12/32)^†^ *Turkish patients*	Gül et al. [8] 10% (11/107)^†^ *Turkish patients*
		≠	≠	≠
		Silingardi et al. [10] 4.5% (8/118)^†^ *Italian patients *	Silingardi et al. [10] 7.4% (2/27)^†^ *Italian patients *	Silingardi et al. [10] 3.8% (5/132)^†^ *Italian patients *

FII G20210A mutation	2-3% Seen only in Caucasians.	Gül et al. [9] 17% (11/64)^†^ *Turkish patients *	Gül et al. [9] 31.3% (10/32)^†^ *Turkish patients *	No data
		≠	≠	
		Silingardi et al. [10] 5.8% (9/118)^†^ *Italian patients *	Silingardi et al. [10] 3.7% (1/27)^†^ *Italian patients *	Silingardi et al. [10] 3.8% (5/132)^†^ *Italian patients *

MTHFR polymorphisms	About 10% are homozygous carriers of the variant C677T.	Karakus et al. [11]: frequency of heterozygosity of C677T was nearly similar between two groups; the homozygosity of C677T was significantly higher in BD patients than healthy controls (*P* = 0.004; OR 5.05, 95% CI: 1.49–17.11). *Study group: 318 patients with BD and 207 healthy controls of Turkish origin*.* * ≠ Ricart et al. [12]: there was no difference between patients and controls in the prevalence of C677T polymorphism. *Study group: 79 patients with BD (23 with thrombosis and 56 withoutthrombosis) and 84 healthy control patients in eastern Spain*.		

Hyperhomocysteinemia (>15 *μ*mol/L)	Levels of homocysteine over 18 *μ*mol/L are associated with an increased risk of thrombosis. Such levels are found in 5%–10%.	Shahram et al. [13] 14.9 ± 13.9 *μ*Mol/L (SD)^‡^ *96 Iranian patients *	Shahram et al. [13] 24.2 ± 13.2 *μ*Mol/L (SD)^‡^ *49 Iranian patients *	Shahram et al. [13] 9.9 ± 6.7 *μ*Mol/L (SD)^‡^ *49 Iranian patients *
			≠	
		No data	Leiba et al. [14] 12.6 ± 3.9 *μ*Mol/L (SD)^‡^ *33 Israeli patients *	No data

Antiphospholipid antibodies	1–5% Usually detectable in low titres.	Tokay et al. [15]: the frequency of IgG and IgM anticardiolipin antibodies (aCL) was 2.4% in BD, 50% in systemic lupus erythematosus (SLE), and 5.6% in healthy controls. No association was found between aCL titres and vascular involvement in BD. *Study group: 128 patients with BD, 20 patients with SLE, and 143 healthy control patients of Turkish origin*. ≠ Hull et al. [16]: statistically significant presence of aCL was found in 13/70 patients with BD and was connected with vascular pathology (8/13). *Study group: 70 patients with BD. Forty originated from Italy, 19 from the United Kingdom, 10 from Middle Eastern countries, and one from the West Indies*.		

^†^Percentage and number of the patients respectively, ^‡^(SD): standard deviation.
